# Antimicrobial Effects of Formulations of Various Nanoparticles and Calcium Hydroxide as Intra-canal Medications Against Enterococcus faecalis: A Systematic Review

**DOI:** 10.7759/cureus.70382

**Published:** 2024-09-28

**Authors:** Seema H Bukhari, Dax Abraham, Shakila Mahesh

**Affiliations:** 1 Conservative Dentistry and Endodontics, Manav Rachna Dental College, Faridabad, IND; 2 Microbiology, Manav Rachna Dental College, Faridabad, IND

**Keywords:** calcium hydroxide, colony-forming units, enterococcus faecalis, nanoparticles, root canal treatment

## Abstract

The combination of calcium hydroxide (Ca(OH)₂) and nanoparticles (NPs) offers a promising approach to improving the efficacy of intra-canal treatments. Their synergistic effects can enhance antimicrobial action, improve penetration, and promote better healing outcomes in endodontic therapy. This review article examines the antimicrobial efficacy of various nanoparticles combined with Ca(OH)₂ against *Enterococcus faecalis* (*E. faecalis*) compared to Ca(OH)₂ alone as an intra-canal medicament. The analysis is based on in vitro studies involving bacterial inoculation on human-extracted teeth. Publications from 2013 to 2024 were retrieved from databases such as PubMed, Scopus, the Cochrane Library, and EBSCOhost and were screened according to specific inclusion criteria. Ultimately, 11 studies met these criteria for inclusion in the systematic review. A meta-analysis was not conducted due to the heterogeneity of the studies regarding the duration of medicament application, analytical methods, and result interpretations. The results indicate that NPs combined with calcium hydroxide exhibit superior bactericidal effects compared to Ca(OH)₂ alone, suggesting their potential as effective intra-canal medicaments.

Thus, a systematic review concluded that nanoparticle-based Ca(OH)₂ intra-canal medicaments exhibit superior antibacterial/antimicrobial capabilities against *E. faecalis* when compared to Ca(OH)₂individually.

## Introduction and background

Endodontic therapy aims to eliminate microflora from the root canal system (RCS). Eliminating bacteria from the root canals has been difficult mainly because of the complexity of the root canal anatomy and the formation of microbial biofilm [1]. However, the complex anatomy of root canals and microbial biofilms present within root canals will significantly challenge successful disinfection. Calcium hydroxide (Ca(OH)₂) often fails to eradicate bacteria such as *Enterococcus faecalis* (*E. faecalis*), a common pathogen associated with endodontic infections due to its resilience and ability to form biofilms [2, 3].

Calcium hydroxide has long been a cornerstone in endodontic treatment due to its property to neutralize bacteria and promote tissue healing. Despite its use, its effectiveness is limited due to inadequate penetration into dentinal tubules and reduced activity against biofilms [4]. To overcome these limitations, researchers have explored the enhancement of Ca(OH)_2_ with nanoparticles (NPs). These tiny particles are engineered at the nanoscale levels with properties that can significantly alter the behavior of the host material [5].

Nanoparticles, including silver, bioactive glass, chitosan-propolis, cerium oxide, and polylactic-co-glycolic acid (PLGA), have antimicrobial properties and the potential to improve the performance of endodontic medicaments. Bioactive glass has excellent regenerative and antimicrobial properties; silver (Ag) is mainly used for its antimicrobial properties. The rate of silver ion release determines its unique antibacterial properties [4, 6]. Nanoparticles of chitosan were used as intra-canal medicaments because of their enhanced antimicrobial action [7,8]. These nanoparticles can offer improved penetration, enhanced antimicrobial activity, and better disruption of biofilms, potentially overcoming the shortcomings of traditional Ca(OH)_2_ treatments.

This systematic review aims to evaluate the effectiveness of NP-based Ca(OH)_2_ formulations for the eradication of *E. faecalis* in root canals. By comparing NP-based treatments with conventional Ca(OH)_2_, the review seeks to provide a comprehensive assessment of their efficacy in improving antimicrobial outcomes, biofilm disruption, and overall endodontic success [9]. The findings of this review could significantly impact clinical practices, guiding the development of more effective endodontic treatments and offering insights into future research directions [10]. The success of endodontic treatment depends on chemo-mechanical disinfection that eliminates the vital or necrotic pulp tissue, kills microorganisms in the RCS, and disrupts microbial biofilm [11].

## Review

Material and methods

Protocol Registration

The Population, Intervention, Comparison, Outcome, and Study design (PICOS) criteria were considered while conducting the systemic review. An advanced and current literature search was provided, ensuring no prior systemic reviews on related subjects were published. The Preferred Reporting Items for Systematic Reviews and Meta-Analyses (PRISMA 2020) statement was followed in this systemic review, and the study protocol was registered in the Open Science Framework (OSF) [12]. This review used the ‘Cochrane Handbook for Systematic Reviews of Interventions’ as a guide and followed the PICOS format [2]. Before developing the research question, a group of writers deliberated on the availability, significance, and need for systemic reviews on relevant topics.

Focused Question

Using PICOS, this review question was formulated [12, 13] by using Population (P): permanent human teeth that had been extracted and were infected with *E. faecalis*; Intervention (I): nanoparticle-based Ca(OH)₂ as intra-medicament; Comparison (C): calcium hydroxide, a regularly/traditionally used intra-canal medicament, was employed as the comparison group. The colony-forming unit (CFU) counts, live/dead bacterial count, and zone of inhibition were used to measure outcome (O). Research (S) included experimental in vitro studies evaluating NP-based calcium hydroxide antibacterial activity compared to calcium hydroxide alone. Through mutual consent, a group of authors (SB, DA, and SM) designed the research question by taking into account the availability, significance, and need for systemic reviews on relevant themes. As a result, this review compares the use of Ca(OH)₂ and various NPS as intra-canal medications and includes in vitro research. Thus, the research question is, “Are nanoparticle-based calcium hydroxide intra-canal medicaments effective against *E. faecalis* compared to calcium hydroxide alone?"

Literature Search Strategies

Scopus, PubMed, EBSCOhost, and Cochrane Library were the major search engines for article retrieval. The search strategy comprised text words and mesh terms, which consisted of the following keywords: ‘‘Nanoparticles”, ‘‘*E. faecalis*,” and ‘’Intracanal medicament’’. To search the articles, the combination used was: (Nanoparticle OR ‘‘Nano-particles”) AND (Intracanal medicament) AND (Endodontics OR ‘‘Regenerative Endodontics”) AND e. AND (faecalis) using the All Fields function in PubMed, Scopus, EBSCOhost, Science Direct, Google Scholar, the Title-ABS-Key function in Scopus, and the Advance Search function with a Medical subheading (MeSh) in the Cochrane Library database. The relevant articles were filtered according to the title, abstract, and papers that matched the inclusion criteria.

Inclusion Criteria

Studies performed on extracted permanent human teeth/dentine disks were included to compare the antimicrobial activity of NPs-based Ca(OH)₂ and Ca(OH)₂ alone as an intra-canal medicament against *E. faecalis*.

Exclusion Criteria

In-vivo studies, controlled trials (non-randomized and randomized), studies on non-human teeth, deciduous teeth, case reports, case series, review articles, unpublished papers, commentaries, and letters to the editor were excluded. Thus, this review article compared an intervention (NP-based Ca(OH)₂) with a control group, i.e., Ca(OH)₂ as an intra-canal medicament, and includes in vitro studies. As such, the research question is: Are nanoparticle-based calcium hydroxide intra-canal medicaments effective against *E. faecalis* when compared to calcium hydroxide alone?

Quality Assessment of the Included Studies

**Figure 1:**
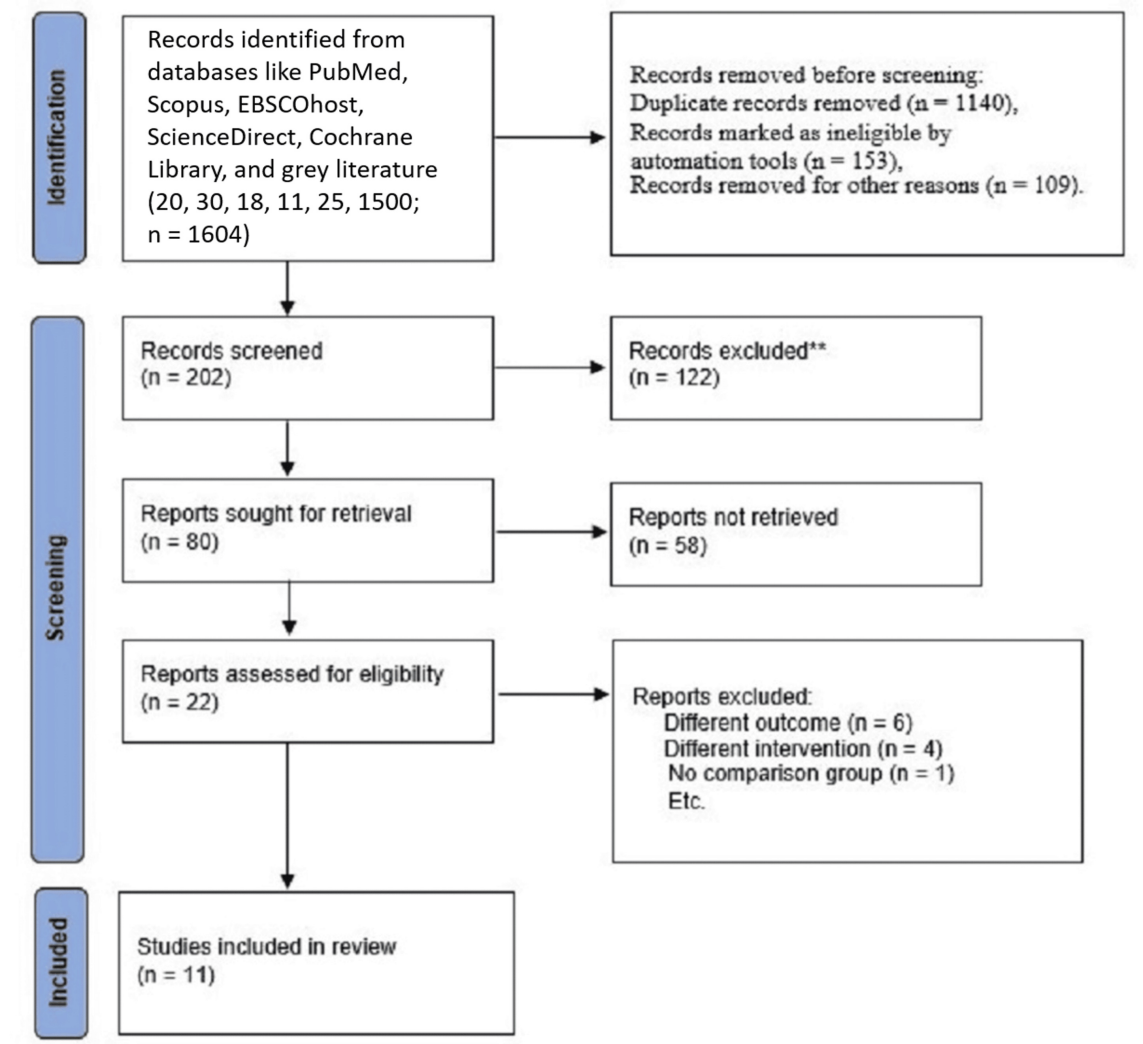
A PRISMA flowchart outlining the study selection process PRISMA: Preferred Reporting Items for Systematic Reviews and Meta-Analysis

Three assessors SB, DA, and SM independently assessed the risk of bias of included study. The systematic review was registered in the Open Science Framework (OSF) and was directed as per the Preferred Reporting Items for Systematic Reviews and Meta-Analysis (PRISMA) 2020 statement as represented in Figure *1*.

Data Collection/Extraction Process

The authors, SB and DA, extracted the data independently, concentrating on the types of studies and related factors. A group of three authors (SB, DA, and SM) then reviewed and finalized the data selection and resolved disagreements through discussion. Data relevant to the inclusion criteria were retrieved, and studies were excluded if data disaggregation was not possible. Data accuracy was confirmed by all three authors.

Results

After the initial database search, 1,604 studies were identified. Following an examination of titles and abstracts, 1,593 studies were excluded. Authors SB and DA removed studies that did not meet the eligibility criteria after duplicates were eliminated. Any uncertainties were resolved through discussion between the reviewers. The remaining 11 studies, which specifically examined the effectiveness of NPs-based Ca(OH)₂ as an intra-canal medication at various time intervals for the removal of *E. faecalis* from the RCS, were included in this systematic review.

Study Characteristics

Table 1 includes 11 studies; seven of them evaluated CFUs by using human-extracted teeth that were inoculated with *E. faecalis*, and three studies evaluated live/dead bacteria via confocal laser microscopy. In contrast, one study checked the zone of inhibition using Muller-Hinton agar. Studies included NPs such as Ag, bioactive glass, chitosan-propolis, polyglycolic acid, cerium oxide, and calcium hydroxide NPs; these are the experimental group in this systematic review, and Ca(OH)_2_ is the control group. One of the three methods was used to quantify the outcome, i.e., bacterial viability by confocal laser scanning microscopy, antimicrobial sensitivity testing (AST), and bacterial culture by counting the CFUs.

**Table 1:** List of outcomes of studies included in systematic review CFUs: colony-forming units; Ca(OH)_2_: calcium hydroxide; NPs: nanoparticles; ADT: agar diffusion test; AgNps: silver nanoparticles

Author, year	Journal	Number of samples	Sample medium	Medicament control group	Medicament test group	Medicament duration as an intra-canal medicament	Outcome measures	Outcome evaluation method		Conclusion
Afkhamiet al. (2022) [13].	Journal of Dental Sciences	24	Human teeth	Calcium hydroxide	Calcium hydroxide with silver nanoparticles	1 day	CFU count	Colonies counted		Studies showed that combining Ca(OH)_2_ with AgNPs even after one day efficiently eliminates *E. faecalis*. Ag-based (CaOH_2_ intra-canal medicament showed significant results in colony count compared to Ca(OH)_2_ alone.
Balto H et.al. (2020) [14].	Journal of Endodontics	90	Dentine disk	Calcium hydroxide	Calcium hydroxide with silver nanoparticles	2 and 4 weeks	Confocal microscopy	Live/dead bacteria		A mixture of Ca(OH)_2_ + AgNPs showed a high antibiofilm effect. A significantly greater proportion of dead cells was observed in the samples treated with Ca(OH)_2_ + AgNPs (90.85% and 98.49%) than those in the samples treated with Ca(OH)_2_ (76.14% and 91.71%) at 2 and 4 weeks, respectively.
Afkahani et.al. (2015) [15].	Journal of Dentistry	54	Human teeth	Calcium hydroxide	Calcium hydroxide with silver nanoparticles	1 week and 1 month	CFU count	Colonies counted		A mixture of calcium hydroxide and AgNPs was the most effective medicament against *E. faecalis* bacteria (*p* < .05), and AgNPs were more effective on the *E. faecalis* biofilm.
Obeid et.al. (2021) [16].	Restorative Dentistry and Endodontics	90	Human teeth	Calcium hydroxide	Bioactive glass nanoparticles	21 days	CFU Count, CLSM	Colonies counted, Live/ dead bacteria		Significant results were obtained when calcium hydroxide was mixed with bioactive glass nanoparticles as compared to calcium hydroxide alone. The CFU for Ca(OH)_2_ is 4.4+0.7×10^4^ cfu/ml, and bioactive glass is 0.9+0.4 ×10^4 ^cfu/ml^ …^highest reduction of CFUs is seen with bioactive glass nanoparticles.
Parolia et al. (2020) [17].	BMC Oral Health	240	Human teeth	Calcium hydroxide	Chitosan-propolis nanoparticle (CPN- 100,CPN-250)	1,3, and 7 days	CFU, CLSM, SEM	Colonies counted Live/dead bacteria.		On days one and three, CPN250 showed a significant reduction of CFUs compared to all other groups (p < .05). SEM images showed root canal dentin treated with CPN250 had less coverage with *E. faecalis* bacteria similarly, CLSM images also showed a higher percentage of dead *E. faecalis* bacteria with CPN250 than to CPN100.
Teja et al. (2023) [18].	BMC Oral Health	400	Human teeth	Calcium hydroxide	Calcium hydroxide with AgNPs, bioactive glass	21 days	CFU	Colonies counted		Ca(OH)_2_ along with nanoparticles showed more effectiveness against *E. faecalis* when compared to the Ca(OH)_2_ alone. CH + BAG S53P4 combination has shown more reduction in the CFU counts. Whereas the CH+chitosan combination was the least effective in reducing the CFU counts among all the combinations
Leelapornpisid et al. (2024) [19].	Australian Society of Endodontology	40	Human teeth	Calcium hydroxide	Calcium hydroxide-loaded poly(lactic-co-glycolic acid) nanoparticles (CH-loaded PLGA NPs)	7 days	CFU	Colonies counted		CH-loaded PLGA NPs demonstrated a significantly lower viable cell than Ca(OH)_2_
Sanju et al. (2022) [20].	Endodontology	80	Human teeth	Calcium hydroxide	1). Cerium oxide nanoparticles(group 2) 2). Combination of cerium oxide nanoparticle and calcium hydroxide(group 3)	1 and 5 days	CFU	Colonies counted		Cerium oxide nanoparticles show a significantly higher antibacterial efficacy when compared to calcium hydroxide and its combination.
Chandra et al. (2017) [21].	Dental hypothesis	250	Human teeth	Calcium hydroxide (group 1).	Silver nanoparticles (AgNP) (group 2) and AgNP with Ca(OH)_2 _(group 3)	1 to 7 days and 7 to 14 days	CFU	Colonies counted		A minimum number of CFUs was found in the AgNP + Ca(OH)_2_ group.
Dianat et al. (2015) [22].	Iranian Endodontic Journal	23	Human teeth	Calcium hydroxide (CH)	Nanoparticle calcium hydroxide	7 days	Agar diffusion test (ADT	Zone of inhibition		Nanoparticle calcium hydroxide with distilled water (DW) produced the greatest inhibition zone in the agar diffusion test, the antimicrobial activity of nanoparticle calcium hydroxide was superior to calcium hydroxide in the culture medium.
Javedi et al. (2014) [23].	Australian Endodontic Journal	66	Human teeth	Calcium hydroxide	Calcium hydroxide with nanosilver particles	1, 7 days	CFU	Colonies counted		The number of CFUs observed with Ca(OH)_2_ + nanosilver as intra-canal medicament was significantly less than that observed with Ca(OH)_2 _alone after 1 or 7 days.

Characteristics of Individual Studies

According to Afkhami et al., the carrier silver nanoparticles (AgNPs) for Ca(OH)2 were most effective against *E. faecalis* biofilm in root canal dentin. A combination of AgNPs with Ca(OH)_2_ exhibited significant residual antibacterial activity against *E. faecalis*. Complete eradication of *E. faecalis* from the RCS cannot be achieved by biomechanical preparation and irrigation, highlighting the necessity of adjunctive intra-canal medicaments. Among the tested agents, combining Ca(OH)₂ and AgNPs emerged as a promising approach, offering superior antibacterial efficacy and sustained activity [13].

An in vitro study conducted by Balto et al. conducted on dentine specimens that were biomechanically prepared and inoculated by *E. faecalis* to establish a three-week-old biofilm model. Specimens in each group were equally subdivided into two subgroups and were incubated for two and four weeks. Prepared medicaments such as Ca(OH)₂ mixed with 0.02% AgNPs, Ca(OH)₂, and AgNPs alone were evaluated to check the antimicrobial efficacy in terms of live/dead bacteria by using confocal microscopy. The study concluded that a combination of Ca(OH)₂ mixed with AgNPs showed greater antibiofilm effectiveness against *E. faecalis* when compared to Ca(OH)_2_ and AGNPs used individually. A significantly greater proportion of dead cells were observed in the samples treated with the combination of Ca(OH)_2_ and AgNPs (90.85% and 98.49%, respectively) compared to the other group of Ca(OH)₂ treated samples (76.14% and 91.71%, respectively) at an interval of two and four weeks, respectively [14].

A study conducted by Afkhami et al. evaluates the effectiveness of NP-based Ca(OH)₂ as an intra-canal medicament in suppressing *E. faecalis *biofilm at one-week and one-month time intervals. Fifty-four extracted single-rooted human teeth were contaminated with *E. faecalis* and divided into four groups, i.e., Ca(OH)₂, Ca(OH)₂ with AgNPs, and Ca(OH)₂ with chlorhexidine. Specimens were analyzed, and colonies were counted after one week and one month. Results concluded that Ca(OH)₂ along with AgNPs was most effective in short-term use, and there were no significant differences after one month against *E. faecalis* bacteria [15].

Obeid et al. conducted a study on the effectiveness of NP-based bioactive glass (BAG) compared to standard BAG, Ca(OH)₂, and saline for eliminating *E. faecalis* biofilms within root canals. After a week of treatment, the BAG-NP group showed the greatest reduction in live bacteria when compared to other treatments. Nano-sized BAG significantly improved antimicrobial efficacy against *E. faecalis* biofilms in root canals. The enhanced performance of BAG-NP suggests that reducing particle size can optimize the antibacterial properties of intra-canal medicament [16].

Another study conducted by Parolia et al. evaluated the antibacterial effect and depth of penetration of chitosan-propolis nanoparticles (CPN) at concentrations of CPN250 and CPN100. The study concluded that CPN250 was effective in reducing *E. faecalis* CFU at depths of 200 μm and 400 μm dentinal tubule on the first and third days. On the seventh day, both CPN250 and CPN100 were equally effective, suggesting CPN250 as an intra-canal medicament for further evaluation [17].

Teja et al. evaluated that Ca(OH)₂, when combined with NPs, was effective against *E. faecalis* compared to Ca(OH)₂ at a three-week interval. The combination of Ca(OH)_2_ and bioactive glass (BAG S53P40) resulted in the most effective, and the combination of Ca(OH)_2_ and chitosan was the least effective in reducing CFU counts. Additionally, Ca(OH)₂ combined with NPs showed greater effectiveness against *E. faecalis* than Ca(OH)₂ individually [18].

Leelapornpisid et al. examined the Ca(OH)₂-loaded PLGA NPs (CH-loaded PLGA NPs) against the biofilms of various microorganisms. Dentine specimens were inoculated by *E. faecalis* in the root canals for three weeks. Following this, the canals (n = 10 per group) were treated with either Ca(OH)₂ or CH-loaded PLGA NPs for seven days. Samples were taken from 0.1 mm dentin of root canals, and CFUs were measured using brain heart infusion (BHI) agar. The results concluded that Ca(OH)₂ and CH-loaded PLGA NPs significantly reduced viable cell counts. Moreover, CH-loaded PLGA NPs showed a markedly lower CFU count compared to Ca(OH)₂ alone (p < 0.001), highlighting its potential as a promising agent for endodontic therapy [19].

A study conducted by Sanju et al. compared the cerium oxide NPs with Ca(OH)₂ against *E. faecalis*. The study involved evaluating the reduction in *E. faecalis* using extracted tooth specimens on the first and fifth days at dentinal depths of 200 μm and 400 μm. The included groups were Ca(OH)₂, cerium oxide NP dispersion, a combination of both, and sterile water. Results concluded that cerium oxide NPs achieved a 66.9% reduction in *E. faecalis* at the 400 μm depth by the fifth day. Cerium oxide NPs showed significantly better antibacterial efficacy [20].

A study by Chandra et al. evaluated the effects of NP-based intra-canal medicaments on dentine specimens at various time points. After 24 hours and seven days, the combination of AgNP and Ca(OH)₂ was more effective than either AgNP or Ca(OH)₂ individually. However, no significant effects were observed at three weeks. The enhanced effectiveness of the combination may be attributed to the ability of *E. faecalis* to withstand the alkaline conditions produced by Ca(OH)₂, and the destruction of the cell wall of the microorganism by AgNP, which disrupts the biofilm and improves the delivery of Ca(OH)₂. After seven days, the AgNP and Ca(OH)₂ combination was more effective against *E. faecalis* when compared to Ca(OH)₂ used alone [21].

Dianet et al. compared the antimicrobial/antibacterial efficacy of NP Ca(OH)₂ with traditional calcium hydroxide Ca(OH)_2_ against *E. faecalis* by using minimum inhibitory concentration (MIC) and agar diffusion tests. The researchers found that NP Ca(OH)₂ was significantly more effective. Nanoparticle Ca(OH)₂ demonstrated a MIC and produced a larger inhibition zone in the agar diffusion test. Additionally, NP Ca(OH)₂ showed greater antimicrobial activity within dentinal tubules at depths of 200 μm and 400 μm compared to Ca(OH)2. These results suggest that NP Ca(OH)₂, due to its smaller particle size and higher surface area, can penetrate deeper into dentinal tubules and better combat *E. faecalis*. Thus, NP Ca(OH)₂ may be a more effective intra-canal medicament for treating root canal infections. More clinical studies are required to confirm these findings and assess NP Ca(OH)₂'s practical application in endodontic therapy [22].

A study by Javidi et al. aimed to evaluate the effectiveness of Ca(OH)₂ and a combination of Ca(OH)₂ with AgNPs in eradicating *E. faecalis* from root canals. Human teeth were infected with *E. faecalis* and treated with 10% Ca(OH)_2_, Ca(OH)_2_ with AgNPs, and sterile water as a control. The number of *E. faecalis* CFUs was measured at one and seven days post-treatment. Results conclude that Ca(OH)₂ with nanosilver significantly reduced CFUs more effectively than Ca(OH)₂ alone at both time points (P < 0.001) [23].

Assessment of Risk of Bias

In the 11 included studies, the risk of bias and methodological quality of the research articles were evaluated using the Office of Health Assessment and Translation (OHAT) tool as shown in Table 2. The tool uses 11 qualities for assessment of the risk of bias, where columns 2 and 3 deal with group allocation and concealment, and columns 4 and 5 of the included study showed a good rating as the study is completely blinded and the characterization of all studies is confident, which deals with columns 6, 7, and 8. All measured outcomes were reported in the form of CFU, live/dead bacteria, and antimicrobial sensitivity tests, and statistical methods were appropriately used for the study and were included in items 9 and 10. Table 1 represents the summary of lists of studies included in the systematic review on the basis of inclusion criteria [24].

Studies that did not report one to three of the items were graded as low-risk, those reported four to six items as moderate bias, and those with more than six non-reported items as having a high risk of bias, as shown in Table 2. The majority of the included studies have a low risk of bias. Although, with the differences in and heterogeneity of the results of the included studies.

**Table 2:** The Office of Health Assessment and Translation (OHAT) tool for Risk of Bias and methodological quality assessment ++: definitely low risk of bias; +: probably low risk of bias; _: probably high risk of bias; NR: insufficient information; _ _: definitely high risk of bias

Vitro-studies	Selection bias		Performance bias		Attrition/Exclusion bias	Detection bias		Selective reporting bias	Other sources of bias
Author, years	Was random allocation present	Was allocation to study groups adequately concealed	Were the experimental conditions identical across study groups?	Were the research personnel blinded to the study group?	Were outcome data complete without attrition or exclusion from analysis	Can we be confident in the intervention characterization	Can we be confident in the outcome assessment	Were all measured outcomes reported?	Statistical methods were appropriate or researchers adhere to the study protocol
Afkhami F et al. (2022) [13]	NR	++	++	NR	++	++	_	++	+
Balto H et.al. (2020) [14]	NR	+	++	_	+	+	_ _	++	++
Afkahani, et al. (2015) [15]	_	++	+	NR	++	+	_ _	+	+
Obeid MF et al. (2021) [16]	NR	+	+	NR	++	++	_	+	++
Parolia A et al. (2020) [17]	C	+	++	_	+	+	_ _	++	++
Teja et al. (2023) [18]	_	++	_	NR	++	++	_	++	++
Leelapornpisid et al.(2024) [19	NR	-	+	NR	++	++	_ _	+	+
Sanju et al. (2022) [20]	NR	++	+	NR	+	+	--	++	+
Chandra et al. (2019) [21]	NR	+	++	-	++	--	-	+	++
Dianat et al (2015) [22]	NR	++	-	NR	+	-	-	++	+
Javedi et al (2015) [23]	NR	+	+	-	+	+	-	+	+

Discussion

The systematic review of 11 studies on NP-enhanced Ca(OH)₂ for eradicating *E. faecalis* from root canals reveals significant advancements in endodontic therapy. The integration of nanoparticles, such as silver, bioactive glass, chitosan-propolis, cerium oxide, and PLGA NPs with Ca(OH)₂ has demonstrated improved antimicrobial properties compared to traditional Ca(OH)₂. This discussion will explore the implications of these findings, their impact on clinical practice, and potential future directions.

Enhanced Antimicrobial Efficacy

A prominent finding across the reviewed studies is the enhanced antimicrobial efficacy of NP-based Ca(OH)₂ formulations. For instance, Afkhami et al. highlighted that Ca(OH)₂ combined with AgNPs exhibited prolonged antibacterial activity against *E. faecalis*, surpassing the effectiveness of Ca(OH)₂ alone. This is corroborated by Javidi et al., who found a significant reduction in CFU with Ca(OH)₂ and nanosilver compared to Ca(OH)₂ used alone. The enhanced performance is attributed to the synergistic effects of nanoparticles, which may disrupt bacterial cell walls and improve the delivery of Ca(OH)₂, thereby increasing its effectiveness [5, 25].

Similarly, studies such as those by Balto et al. and Omid et al. revealed that NPs like AgNPs and BAG significantly reduce bacterial viability and CFU counts in dentinal tubules. These NPs likely enhance the penetration and stability of the medicament, offering superior antimicrobial properties [26]. For example, BAG-NP demonstrated the highest reduction in live bacteria and increased the proportion of dead bacteria compared to traditional treatments.

Biofilm Disruption, Depth of Penetration, and Residual Activity

Biofilm formation by *E. faecalis* is a significant challenge in root canal therapy, as it can protect bacteria from traditional intra-canal medicaments. The studies reviewed show that NP-enhanced root canal treatment outcomes by disrupting biofilms [27, 28]. Balto et al. observed that Ca(OH)₂ combined with AgNPs had a higher antibiofilm effect, with a greater proportion of dead cells in biofilm models when compared to Ca(OH)₂ alone. Teja et al. and Omid et al. suggested nanoparticle-based formulations, such as NCH and BAG-np, penetrate deeper and more effectively within the dentinal tubules than traditional Ca(OH)₂. Nanoparticles' smaller particle size and higher surface area enhance their ability to kill bacteria within dentinal tubules. This improved penetration can lead to more effective disinfection and a reduced risk of persistent infection [28, 29].

Clinical Relevance and Future Directions

The reviewed studies conclude that NP-enhanced Ca(OH)₂ is a promising advancement in endodontic therapy. The enhanced antimicrobial activity, improved biofilm disruption, and deeper penetration make these formulations potentially more effective than traditional Ca(OH)₂ intra-canal medicament [5, 30]. Future research should focus on optimizing NP formulations to balance efficacy, safety, and cost. Investigations into the potential interactions of NPs with other endodontic materials and treatments are also necessary [26].

Recent Advances

Some research employed mesoporous calcium-silicate NPs that were infused with a low dose of silver ions, with Triton X-100 used to regulate the delivery of these AgNPs (referred to as M-AgTx). These particles were effective in eliminating a biofilm of *E. faecalis* that had been established for 28 days [31]. Likewise, M-AgTx showed excellent antimicrobial ability against *E. faecalis* and high substantivity on dentin. Silver NPs with graphene oxide NPs had an almost negligible effect on the root dentin microhardness compared to calcium hydroxide alone or when combined with AgNPs [32, 33].

Green biosynthesis of AgNPs by using plants and fungi represents a promising approach for combating *E. faecalis* infections. This method not only achieves comparable antimicrobial efficacy to traditional agents, such as Ca(OH)₂ but also offers advantages in terms of cost efficiency and reduced risk of toxic byproducts. Fungi, with their simple nutrient requirements and ability to produce particles with uniform size and composition, act as effective "nano-factories." Similarly, plants like *Andrographis paniculata* and *Ocimum sanctum Linn*. also contribute to this efficient synthesis process. Overall, these green methods of NPsynthesis are effective in the treatment of biofilm-associated infections [34].

Combining Ca(OH)₂ with NPs can improve its use as an intra-canal medicament, but several limitations exist. Stability is a key concern, as NPs may clump together, reducing their effectiveness. Additionally, some NPs can be toxic to nearby tissues, which poses risks during treatment. There is also limited research on the long-term effects and interactions of these combinations. Moreover, using NPs can be costly, making them less accessible for routine use. These issues can affect the overall effectiveness of Ca(OH)₂ and NPs in endodontic treatments.

In summary, NPs of Ca(OH)₂ intra-canal medicaments represent a significant advancement in root canal therapy, offering improved antimicrobial properties and enhanced treatment outcomes. Future research and clinical trials will be required to evaluate the potential of these innovative treatments and their role in modern endodontics.

## Conclusions

The challenge posed by *E. faecalis* in endodontic treatments primarily lies in its ability to form resilient biofilms, which complicate effective removal and can lead to treatment failures. Traditional agents like Ca(OH)_2_ often exhibit limited antibacterial efficacy against this pathogen. In contrast, AgNPs present a compelling alternative due to their ability to release silver ions with potent bactericidal and anti-inflammatory properties. Research has shown that AgNPs demonstrate significant antibacterial activity against both *E. faecalis* and *Candida albicans*, with effectiveness comparable to that of Ca(OH)_2_. This suggests that AgNPs could enhance treatment outcomes in root canal procedures, making them a promising candidate for improving the management of infections in endodontics and potentially leading to better healing and reduced complications.

The integration of NPs with Ca(OH)2 intra-canal medicaments significantly enhances their antimicrobial efficacy against *E. faecalis* in root canals compared to traditional Ca(OH)₂ alone. Nanoparticle-enhanced formulations improve biofilm disruption, penetration into dentinal tubules, and overall antimicrobial activity. However, further studies are required to assess the biocompatibility and long-term effectiveness of these advanced treatments in endodontic therapy.

## References

[1] Mohammadi Z, Shalavi S, Yazdizadeh M (2012). Antimicrobial activity of calcium hydroxide in endodontics: a review. Chonnam Med J.

[2] Cosan G, Ozverel CS, Yigit Hanoglu D, Baser KH, Tunca YM (2022). Evaluation of antibacterial and antifungal effects of calcium hydroxide mixed with two different essential oils. Molecules.

[3] Kim D, Kim E (2014). Antimicrobial effect of calcium hydroxide as an intracanal medicament in root canal treatment: a literature review - part I. In vitro studies. Restor Dent Endod.

[4] Hussein F, Imam H (2022). The effect of eggshell and seashell nanoparticles alone and combined with Nd: YAG laser on occlusion and remineralization potential of patent dentinal tubules: an In vitro study. J Lasers Med Sci.

[5] Özdemir O, Kopac T (2022). Recent progress on the applications of nanomaterials and nano-characterization techniques in endodontics: a review. Materials (Basel).

[6] Bonilla-Represa V, Abalos-Labruzzi C, Herrera-Martinez M, Guerrero-Pérez MO (2020). Nanomaterials in dentistry: state of the art and future challenges. Nanomaterials (Basel).

[7] Ghadi A, Mahjoub S, Tabandeh F, Talebnia F (2014). Synthesis and optimization of chitosan nanoparticles: potential applications in nanomedicine and biomedical engineering. Caspian J Intern Med.

[8] Dako T, Pop M, Fulop J, Kantor J, Monea M (2020). The use of calcium hydroxide as an intracanal medicament in the treatment of large periapical lesions. A review. Acta Medica Transilvanica.

[9] Prashanth BR, Revankar B, Karale R, Moogi PP, Mangala MG, Sahoo AK (2024). Comparative assessment of nanosized intracanal medicaments on penetration and fracture resistance of root dentin - an in vitro study. J Conserv Dent Endod.

[10] Iandolo A (2023). Modern therapeutic strategies in endodontics and restorative dentistry. Medicina (Kaunas).

[11] Oncu A, Huang Y, Amasya G, Sevimay FS, Orhan K, Celikten B (2021). Silver nanoparticles in endodontics: recent developments and applications. Restor Dent Endod.

[12] Page MJ, McKenzie JE, Bossuyt PM (2021). Updating guidance for reporting systematic reviews: development of the PRISMA 2020 statement. J Clin Epidemiol.

[13] Afkhami F, Rostami G, Batebi S, Bahador A (2022). Residual antibacterial effects of a mixture of silver nanoparticles/calcium hydroxide and other root canal medicaments against Enterococcus faecalis. J Dent Sci.

[14] Balto H, Bukhary S, Al-Omran O, BaHammam A, Al-Mutairi B (2020). Combined effect of a mixture of silver nanoparticles and calcium hydroxide against Enterococcus faecalis biofilm. J Endod.

[15] Afkhami F, Pourhashemi SJ, Sadegh M, Salehi Y, Fard MJ (2015). Antibiofilm efficacy of silver nanoparticles as a vehicle for calcium hydroxide medicament against Enterococcus faecalis. J Dent.

[16] Obeid MF, El-Batouty KM, Aslam M (2021). The effect of using nanoparticles in bioactive glass on its antimicrobial properties. Restor Dent Endod.

[17] Parolia A, Kumar H, Ramamurthy S, Davamani F, Pau A (2020). Effectiveness of chitosan-propolis nanoparticle against Enterococcus faecalis biofilms in the root canal. BMC Oral Health.

[18] Teja KV, Janani K, Srivastava KC (2023). Comparative evaluation of antimicrobial efficacy of different combinations of calcium hydroxide against Enterococcus faecalis. BMC Oral Health.

[19] Leelapornpisid W, Wanwatanakul P, Mahatnirunkul T (2024). Efficacy of calcium hydroxide-loaded poly(lactic-co-glycolic acid) biodegradable nanoparticles as an intracanal medicament against endodontopathogenic microorganisms in a multi-species biofilm model. Aust Endod J.

[20] Sanju D, Kumari VA, Thomas T, Thomas JT, Sujeer R (2022). Comparative evaluation of cerium oxide nanoparticles and calcium hydroxide as intracanal medicament against Enterococcus faecalis on tooth substrate: an in vitro study. Endodontology.

[21] Chandra A, Yadav RK, Shakya VK, Luqman S, Yadav S (2017). Antimicrobial efficacy of silver nanoparticles with and without different antimicrobial agents against Enterococcus faecalis and Candida albicans. Dent Hypotheses.

[22] Dianat O, Saedi S, Kazem M, Alam M (2015). Antimicrobial activity of nanoparticle calcium hydroxide against enterococcus faecalis: an in vitro study. Iran Endod J.

[23] Javidi M, Afkhami F, Zarei M, Ghazvini K, Rajabi O (2014). Efficacy of a combined nanoparticulate/calcium hydroxide root canal medication on elimination of Enterococcus faecalis. Aust Endod J.

[24] Ho BV, van de Rijt LJ, Weijenberg RA, van der Maarel-Wierink CD, Lobbezoo F (2022). Oral health assessment tool (OHAT) deputized to informal caregivers: go or no go?. Clin Exp Dent Res.

[25] Miglani S, Tani-Ishii N (2021). Biosynthesized selenium nanoparticles: characterization, antimicrobial, and antibiofilm activity against Enterococcus faecalis. PeerJ.

[26] Eskandari F, Abbaszadegan A, Gholami A, Ghahramani Y (2023). The antimicrobial efficacy of graphene oxide, double antibiotic paste, and their combination against Enterococcus faecalis in the root canal treatment. BMC Oral Health.

[27] Raura N, Garg A, Arora A, Roma M (2020). Nanoparticle technology and its implications in endodontics: a review. Biomater Res.

[28] Roig-Soriano X, Souto EB, Elmsmari F (2022). Nanoparticles in endodontics disinfection: state of the art. Pharmaceutics.

[29] Sharifi R, Vatani A, Sabzi A, Safaei M (2024). A narrative review on application of metal and metal oxide nanoparticles in endodontics. Heliyon.

[30] Wong J, Zou T, Lee AH, Zhang C (2021). The potential translational applications of nanoparticles in endodontics. Int J Nanomedicine.

[31] Nasim I, Jabin Z, Kumar SR, Vishnupriya V (2022). Green synthesis of calcium hydroxide-coated silver nanoparticles using Andrographis paniculata and Ocimum sanctum Linn. leaf extracts: an antimicrobial and cytotoxic activity. J Conserv Dent.

[32] Bruniera JF, Gabriel-Silva L, Goulart RS, Silva-Sousa YT, Lara MG, Pitondo-Silva A, Miranda CE (2020). Green synthesis, characterization and antimicrobial evaluation of silver nanoparticles for an intracanal dressing. Braz Dent J.

[33] Duan M, Fan W, Fan B (2021). Mesoporous calcium-silicate nanoparticles loaded with low-dose triton-100+ag+ to achieve both enhanced antibacterial properties and low cytotoxicity for dentin disinfection of human teeth. Pharmaceutics.

[34] Halkai KR, Mudda JA, Shivanna V, Rathod V, Halkai R (2018). Evaluation of antibacterial efficacy of fungal-derived silver nanoparticles against Enterococcus faecalis. Contemp Clin Dent.

